# CO_2_ Transoral Laser Microsurgery in Benign, Premalignant and Malignant (Tis, T1, T2) Lesion of the Glottis. A Literature Review

**DOI:** 10.3390/medicines6030077

**Published:** 2019-07-22

**Authors:** Carlos Miguel Chiesa-Estomba, Jose Angel González-García, Ekhiñe Larruscain, Christian Calvo-Henríquez, Miguel Mayo-Yáñez, Jon A Sistiaga-Suarez

**Affiliations:** 1 Otorhinolaryngology—Head & Neck surgery Department, Hospital Universitario Donostia, 20014 San Sebastian—Donostia, Spain; 2 Biodonostia Health Research Institute, 20014 San Sebastian—Donostia, Spain; 3 Otorhinolaryngology—Head and Neck Surgery Department, Complexo Hospitalario Universitario Santiago de Compostela (CHUS), 15706 Santiago de Compostela, Spain; 4 Clinical Research in Medicine, International Center for Doctorate and Advanced Studies (CIEDUS), Universidade de Santiago de Compostela (USC), 15782 Santiago de Compostela, Spain; 5 Otorhinolaryngology—Head and Neck Surgery Department, Complexo Hospitalario Universitario A Coruña (CHUAC), 15006 A Coruña, Spain

**Keywords:** glottis, CO_2_, laser, surgery

## Abstract

Carbon Dioxide transoral laser microsurgery represents a reliable option for the treatment of early glottic carcinoma (Tis–T2), with good functional and oncological outcomes, nowadays representing one of the main options in larynx preservation protocols. The development and improvement of laser devices means surgeons are able to use more precise instruments compared with classic cold dissection in laser-assisted phonosurgery. Secondary effects on voice, swallowing, or quality of life as well as complications have been well documented. Also, with the introduction of a new proposal for staging systems following the principle of the three-dimensional map of isoprognostic zones, the use of narrow-band imaging in clinical evaluation and intraoperative, and the implementation of diffusion-weighted magnetic resonance during preoperative evaluation, the development of new tools to improve surgical quality and preliminary reports regarding the use of carbon dioxide laser in transoral robotic surgery suggests an exciting future for this technique.

## 1. Introduction

Laryngeal squamous cell carcinoma (LSCC) is a common malignancy, representing 30%–50% of all neoplasms in the head and neck, with 157,000 new cases diagnosed worldwide in 2012 [1]. The management of early stage (Tis, T1, T2) laryngeal cancer (Supraglottic, Glottic, and Subglottic) has evolved over the last 40 years from the classic open surgical resection techniques to a less aggressive and more functional endoscopic approach due to the evolution of surgical strategies, diagnostic workup, and follow-up protocols. Nowadays, local control and laryngectomy-free survival in glottic cancer (T1–T2) following transoral organ preservation surgery using CO_2_ Laser can be successful in more than 80%–90% when in experienced hands [2]. However, the main advantage of transoral laser microsurgery remains in the possibility of minimizing the area of the larynx, which is resected during the surgery, preserving more healthy tissue and optimizing the patient’s recovery, airway management, as well as post-surgical voice without compromising their overall survival [3].

CO_2_ Laser Surgery of the Larynx has gained followers around the world during these four decades, becoming the gold standard for almost all early stage cancer (Tis–T2). This is the reason why in this review, the authors try to summarize and expose the most relevant concepts about the technique.

## 2. General Aspects and Physical Properties

The term LASER is an acronym for *Light Amplification by Stimulated Emission of Radiation*. This represents a different kind of light due to its properties. Light produced by laser differs from ordinary light because it is monochromatic (all photons have the same color and the same wavelength), collimated (all photons travel in a parallel fashion), and coherent (travels in the same direction and synchronously).

The CO_2_ is contained in gas state in a cavity. The deposit has one end with a 100% mirror, while at the other end, a small proportion of the light is transmitted and escapes through a partially reflecting mirror. The largest portion is reflected again into the system and continues the laser process. The energy of the laser is usually measured and the main variables in laser functionality are: Wavelength, Energy, Spot Size, and Spectrum.

Due to its wavelength (10,600 nm), the CO_2_ Laser is absorbed by water, allowing very predictable cutting and an ablation mode with an optical penetration that is limited to tissues. Moreover, vessels smaller than 0.5 mm are coagulated, meaning bleeding is able to be regulated, providing an excellent view during surgery. 

## 3. Brief History of CO_2_ Laser of the Larynx

At the beginning of the 1970s, due to the development of the equipment and techniques, Prof. Geza Jako started to apply the CO_2_ laser energy in the larynx of cadavers. In 1972, Jako published an experimental study about the use of a carbon dioxide laser in the larynx of 13 dogs [4]. Some of the most relevant contributions of this paper are related to the importance of the depth of penetration, which could be controlled by appropriate radiation dosage and the possibility of limiting the amount of tissue affected. Moreover, he describes the formation of smoke during the procedure, disturbing the visual field and also the risk of damage to the trachea. 

In the same year, Strong and Jako were the first to publish the use of CO_2_ laser, in combination with microscopic visualization and direct laryngoscopy, for the surgical treatment of benign laryngeal lesions in human patients [5].

In 1975, Strong presented a detailed description of the laser excision of vocal fold carcinoma in 11 patients (dissection method, suction, laser set-up). This was the first description of a laser resection due to malignant pathology and represents the start point for the future development of this technique [6]. Three years later, Vaughan, Strong, and Jako presented a series of 31 patients affected by laryngeal carcinoma using the CO_2_ laser, although not all of them with curative intent [7].

In the 1980s, the first reports from European centers were published [8,9]. Steiner, in 1980, and Annyas, in 1984, published the first large series including the results of more than 50 patients treated using the CO_2_ laser in both papers [10,11]. However, Annyas was concerned about the poor functional results of the surgical approach in comparison with patients who were treated with radiotherapy. These studies represent the beginning of the spread of the technique across the continent. In the following years, different improvements in the illumination method or microscope magnification, the development of different microspot manipulators, allowing the surgeon to make limited resections and differentiate healthy tissue from affected tissue, preserving disease-free adjacent areas, and even cutting through the tumor whenever needed without compromising the overall survival, made this kind of treatment popular. This concept, introduced and popularized by Steiner, was supported by a long list of publications, making it possible to extend the use of the laser in the upper aerodigestive tract in the following years, improving the functional and oncological results of this technique and changing some major oncological paradigms. 

In 1998, Fink et al. showed that multilayered dielectric mirrors could be constructed with omnidirectional polarization-independent reflectivity [12]. This kind of technology was used to create hollow fibers that can handle the surgical levels of CO_2_ laser input power (Omniguide Communications, Inc, Cam- bridge, Massachusetts) and in 2006, Zeitels et al. assessed the suitability of this technology for transoral endolaryngeal partial laryngectomy procedures in a canine model, being the starting point to develop more precise indications for laser [13].

## 4. Classification Systems

The absence of uniformity between the different classification proposed by some authors at the beginning render the comparison and pooling of results impossible. For this reason, 2000 surgeons from the working committee of the European Laryngological Society (ELS) proposed a classification for cordectomies based on some criteria, like the depth and extent of surgical resection not exclusive for CO_2_ laser surgery but widely used by laser surgeons [14]. In 2007, the classification was revised, and the anterior commissure resection was included [15]. And due to the success of this method, in 2009, the same committee proposed a classification of endoscopic supraglottic laryngectomies [16]. Thanks to this classification system, results from different departments around the world could be compared, obtaining a better understanding of the benefits of this technique [2] (Table 1).

More recently, members of the Working Committee for Nomenclature of the ELS proposed a classification and definition of the different types of procedures, performed transorally or transnasally, emphasizing the type of laser used as well as the way the laser is transmitted. In this consensus, what is usually called TLM, surgical procedures performed with laser are defined as CO_2_ laser transoral microsurgery or CO_2_TOLMS [17].

## 5. Evidence in Benign and Premalignant Lesions

**Figure 1:**
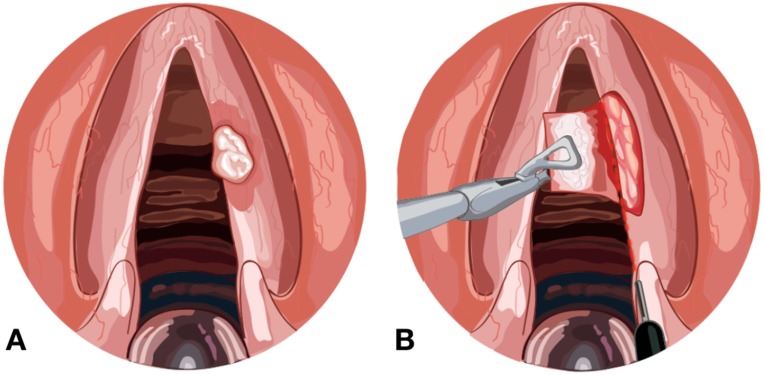
(**A**) Leukoplakia in the right vocal fold. (**B**) CO_2_ laser beam delivered through a flexible hollow tube to remove the lesion, preserving the ligament.

The development and improvement of the micro-spot and micromanipulator, meaning surgeons are able to use a more precise instrument compared with cold dissection, has promoted the development of “Laser-assisted phonosurgery”, a term used to describe all the approaches in which a benign or premalignant lesion of the larynx is treated using the CO_2_ Laser (Figure 1). Recently, a CO_2_ laser beam delivered through a flexible hollow device has become available, which delivers the energy close to the target and allows the surgeon to perform a more precise excision [18].

Table 2 shows the different types of lesion (Benign, Acquired, or Pre-Malignant) that can be treated with this technique. It must be noted that it is important to modify the parameters in the machine to improve our functional results and to avoid thermal damage to the surrounding healthy tissue. 

Therefore, some technical recommendations have been described, like the use of hydrodissection before resection in order to protect the vocal ligament [19] due to the high content of fibroblast in the lamina propia, which may predispose it to major scarring after surgical trauma [20]. Also, the creation of a microflap to protect the vocal ligament after resection like in the treatment of Reinke edema or the use of a high-power pulsed mode to reduce the thermal effect and to improve the consequent coagulation of the area surrounding the surgical site and improving the thermal relaxing time allows the tissue to cool down [21].

CO_2_TOLMS is also an option in the treatment of subglottic hemangioma and the first case described using this technique dates back to 1979. A success rate of 88.9%–100% and a low complication rate (subglottic stenosis) was described by different authors [22,23,24,25]. Nicolai et al. found the CO_2_ laser superior to Nd-YAG laser in a series with 14 and 17 patients, respectively. In this series, no subglottic stenosis was reported versus 15.4% in the Nd-YAG group [26]. 

## 6. Evidence in Malignant Pathology

### 6.1. CO_2_TOLMS in Early Larynx Cancer (Tis,T1,T2)

**Figure 2:**
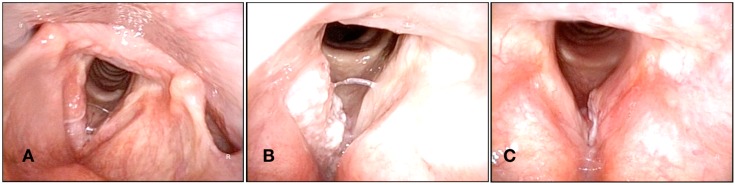
(**A**) Superficial lesion in the middle third of the right vocal fold. (**B**) Ulcerative-exophytic lesion involving all the right vocal fold. (**C**) Lesion affecting the anterior third of the left vocal fold. All of them were positive for malignancy and treated using CO_2_TOLMS.

Regarding results from different studies published about the treatment of early larynx cancer, Lucioni et al. report a local control of 90% for T1a and 81% for T1b with primary laser. Being the ultimate local control with laser alone 98% and 90%, respectively, and larynx preservation rate being 98% in both [27]. Canis et al. published the 27-year experience (1979–2006) including 404 cases of T1a glottic carcinoma, reporting a local control rate of 86% and larynx preservation rate of 97%, with a disease-specific survival of 98% [28]. Regarding T2 tumors, Canis et al. published their data about the treatment of T2 (1979–2006) divided into 142 T2a (mobile cord) and 127 T2b (fixed cord). The 5-year local control was 76% for T2a tumors and 57% for T2b tumors. Larynx preservation was 93% in T2a patients and 83% in T2b patients, with a disease-specific survival of 93% and 84%, respectively [29]. Chiesa-Estomba et al. reported a 79.3% local control, with just one surgery including T1a, T1b, and T2 tumors reaching a 98.3% local control after a second look intervention [30]. These results are similar to those described in the literature by Lee et al., who described an exclusive local control with the laser in 94.2% of patients [31]. In 2013, O’Hara published a review of 16 studies about local control after CO_2_TOLMS and reported a rate of local control for T1a (88%, n = 985), but found that T1b tumors have a lower local control rate, although the study population in this study was also smaller (78%, n = 187) [32] (Figure 2).

### 6.2. Anterior Commissure

The anterior commissure (AC) involvement was considered to be a risk factor for recurrence by some authors [33,34,35,36], and for others, it was not [37,38,39,40]. In 2009, Rödel published an extensive analysis including 463 patients, showing the relation between the involvement of the AC and the decreased local control in T1a and T1b, but not in T2 tumors, without demonstrated compromise to their survival. The author discusses that CO_2_TOLMS is still an effective treatment for tumors located in the AC, as most of the recurrent small tumors could still be treated with further CO_2_TOLMS procedures [41]. 

Data published by Hakeem found local control significantly lower in T2 patients with AC involvement in contrast to those patients treated by T1a or T1b tumors, although there was a trend for poorer local control in T1b tumors [42]. Laryngeal preservation rate and overall survival were not affected. Moreover, Hoffman found patients with Tis–T2 glottic carcinoma and AC involvement to have lower local control, laryngeal preservation rate, and disease-specific survival [43].

As highlighted by Peretti, when a tumor in the anterior commissure is evaluated, it is essential to assess the extension in the horizontal plane or in the vertical plane affecting the supraglottis or the subglottis, being that those tumors with pure horizontal spread a good indication for CO_2_TOLMS [44]. The major risk of poorer local control arises from tumors with vertical extension from the anterior commissure and is related to the close relationship of the inner larynx with the underlying visceral spaces (pre-epiglottic space and subglottis) carrying the risk of microscopic spread into these areas [37]. 

At the level of true vocal folds, thyroid cartilage is situated directly underneath the vocal folds, which is attached to the cartilage by the Broyles’ ligament, which is formed by the union of the thyroepiglottic ligament and the attachment of the vocal ligaments. This structure consists of a dense fibroelastic tissue without glandular structures, blood, or lymphatic vessels [37]. Across the literature, this structure is considered to be a weak point by some authors where tumors can easily penetrate the cartilage, transforming a T1 tumor into a T4 [43,45]. Conversely, others authors consider that the Broyles’ ligament protects the cartilage and this is the reason why tumors with superficial extension in the AC, especially in the horizontal plane (T1), rarely show thyroid cartilage infiltration [37,46,47]. 

### 6.3. Subglottal Extension 

A primary subglottic carcinoma is uncommon, and these lesions are generally related to the caudal spreading below the free margin of the true vocal folds [48]. Those lesions with more than 1 cm of subglottic extension of more than 1 cm (T2) should be considered as moderately advanced and in those few cases, when we consider treating using CO_2_TOLMS, adjuvant therapy should be considered.

### 6.4. Resection Margins 

The assessment of pathological specimens is a specific problem in CO_2_TOLMS because of small sample size, carbonization, retraction, and difficult orientation. It is generally accepted that some patients with positive or inadequate margins will not develop a recurrence. Also, the relationship between resection margin status and relapse is still unclear. 

#### 6.4.1. Margin Size

According to recommendations at the glottic level, 1–2 mm can be considered an adequate margin. However, some authors suggested that margins less than 0.5 mm may be enough in Tis–T1 carcinoma due to the fact that the ligament still forms a barrier, which prevents the tumor spreading in early lesions. When the tumor has spread beyond the ligament (T2), authors like Canis et al. recommend margins of 2–3 mm [29]. 

#### 6.4.2. How to Classify Margin Involvement 

To classify the degree of involvement of surgical margins, the classification proposed by Blanch et al., who aside from setting margins as positive or negative, also included the presence of uncertain margins, is recommended [49].

#### 6.4.3. Handling of Positive or Inadequate Margins

In these cases, some authors prefer a wait and see policy [50,51,52,53]. However, other authors prefer to first perform a second-look procedure [54,55,56] and other authors recommend re-treatment with CO_2_TOLMS or radiotherapy [57,58,59]. It is important to consider that the use of the CO_2_TOLMS evaporation mode in the surgical wound bed after specimen resection adds approximately 0.05–0.5 mm to the surgical margin [50,52,54].

#### 6.4.4. Recurrence Rate

Those authors who propose a wait and see policy report recurrence rates of 13.5%. However, those who choose a more aggressive approach and re-treat most patients report a recurrence rate of 9%. However, Sigston et al. found that 84% of their patients with positive margins would potentially have received unnecessary additional treatment without this wait and see policy [50]. One of the main aspects in CO_2_TOLMS remains in the surgeon experience, allowing the surgeon to offer an intra-operative opinion on how radical the surgery was, being that this is one of the most important factors in decision-making. Moreover, earlier symptoms, such as dysphonia, and the accessible localization of glottic lesions make follow-up adequate for monitoring patients. More recently, Hendriksma et al. showed that additional wound bed biopsies can help predict recurrence after TLM in early glottic carcinoma in which ELS type I–III resection was performed and can help to identify those patients where additional treatment is indicated [60].

#### 6.4.5. Second Look

ELS recommendations during the follow-up of patients after treatment for laryngeal cancer recommend a second-look in case of positive margins which can be indicated in other situations such as close margins, granulomas, and web formation, or in those cases with involvement of the anterior commissure, the laryngeal ventricle, or the subglottis [61].

### 6.5. Salvage CO_2_TOLMS

Some authors suggest the use of CO_2_TOLMS in the treatment of recurrent disease either after initial laser resection or after RT. However, evidence related to local control in recurrent disease is lower than in primary surgery. Rödel et al. reported 5-year local control and larynx preservation rates of 64% and 91%. The authors performed salvage CO_2_TOLMS in patients with an early recurrence when the patient was initially treated for early glottic carcinoma (pTis–T2), concluding that in the case of local recurrence after primary CO_2_TOLMS for early glottic carcinoma, a salvage CO_2_TOLMS seems to be an option as an organ-preserving treatment in some cases. In contrast, they recommend salvage laryngectomy in patients with advanced local or loco-regional recurrence [62]. 

In a study performed by Peretti et al., salvage CO_2_TOLMS was possible in 86% of patients where the recurrence affected the same area, and in 74% if the recurrence affected an adjacent area with superficial spreading or multifocal distribution, being those patients with submucosal recurrence with spread into visceral spaces of the larynx or cartilaginous framework infiltration, not cured endoscopically [37]. Huang et al. analyzed 50 patients with rT1–T2 lesions treated with salvage CO_2_TOLMS; in this cohort, local control was possible in 70% of the patients with a larynx preservation of 88% and disease-specific survival of 98%. However, a second recurrence after salvage CO_2_TOLMS significantly impacted larynx preservation rates and disease-specific survival [63]. 

## 7. Functional Outcomes After Laser Surgery of the Larynx

As an organ preservation surgery, besides a proper oncological result, a good functional outcome is the second main objective of CO_2_TOLMS. Usually these functional advantages can be attributed to the more conservative nature of an endoscopic procedure because healthy tissue is not involved during the approach, being that this is the most relevant difference when comparing with open procedures where the skin as well as the thyroid cartilage and the soft tissues (Strap muscles) are involved. The incision and posterior suturing of these structures will almost always result in airway swelling and the need for tracheotomy being avoided in most endoscopic resections [64].

In this way, the three most relevant factors should be the voice outcome, deglutition outcome, and the need for tracheostomy.

### 7.1. Voice Outcome

Voice is undoubtedly one of the main factors to take into consideration when we treat a patient affected by a laryngeal tumor in the early stages. In the treatment of early larynx carcinoma, both radiotherapy and CO_2_TOLMS provide high and comparable curable rates and voice outcomes. However, there are several advantages to the use of CO_2_TOLMS, such as being a one-session treatment almost in the majority of early cases, repeatable, cost-effective, associated with reduced morbidity, and a short hospitalization time. 

Vilaseca et al., in a prospective study that included patients treated for early and locally advanced carcinoma, obtained favorable results regarding the improvement in quality of life after surgery for most of their subjects and speech preservation, again, being the factor assessed [65]. Van Gogh et al., in a prospective study, evaluated the voice outcome in patients treated with RT or laser surgery in T1a carcinomas. Their results showed that vocal recovery was faster in the group of patients treated with laser. Three months after surgery, they did not find differences for normal voice, except for fundamental frequency and at the highest pitch. After the analysis, the authors concluded that CO_2_TOLMS should be the treatment of choice for T1a tumors [66]. However, Remmelts et al. found evidence against the use of CO_2_TOLMS in patients with deeply infiltrative T1a lesions and T1b lesions, since voice quality might become worse when a laser resection is performed and the vocal ligament or the muscle is affected [67].

More recently, Greulich et al. conducted a meta-analysis about the voice outcomes following radiation therapy versus CO_2_TOLMS for T1 glottic carcinoma and concluded that Voice Handicap Index (VHI) scores were comparable following RT and CO_2_TOLMS for T1 glottic carcinoma, suggesting no difference in the functional voice outcomes between the treatment types [68].

### 7.2. Swallowing Outcome

Deglutition outcome is directly related to the need of a nasogastric feeding tube in the postoperative period. Sometimes this can be necessary during a short period of time, especially in supraglottic tumors. However, it is very rare in glottic tumors. Results after CO_2_TOLMS can be worse in patients exposed to radiation therapy or after neck dissection due to lymphatic stasis. Moreover, in patients treated by CO_2_TOLMS of the supraglottis, in whom swallowing rehabilitation could be prolonged over time, a temporary gastrostomy may be recommended. 

Most authors have suggested that there is better recovery of swallowing after CO_2_TOLMS. These outcomes could be due to the less invasive nature of CO_2_TOLMS, where the muscular structures involved in swallowing are usually preserved, whereas open surgery involves damage to the strap muscles and modification of anatomical structures and, therefore, the alteration of swallowing mechanics [69]. Other parameters can be correlated with the outcome of swallowing after surgery, like T classification or psychological motivation [64].

Overall, <5% of patients will suffer an aspiration pneumonia after a CO_2_TOLMS, and almost all of them are related to supraglottic tumor resection. In these cases, it is important to be aware and promote early deglutition rehabilitation to avoid the risk of microaspiration, especially in patients who are older than 65 years [70].

### 7.3. Tracheostomy 

A tracheostomy is seldom needed after CO_2_TOLMS in early tumors. This can be an option when a bulky tumor makes it impossible to perform an oral intubation, when the airway must be protected after an extended resection, when a patient suffers a massive bleeding, or when the patient develops dyspnea after surgery secondary to edema. However, this limits the restoration of normal swallowing, a normal due to fixation of the larynx, and limitation of its natural movements. In almost all cases, the tracheostomy is just temporary. 

## 8. Quality of Life

One of the widely accepted advantages of CO_2_TOLMS for early glottic carcinoma is that the quality of life (QoL) is good and usually does not differ significantly from healthy controls after surgery. Regarding this topic, there are two important papers published by Bernal-Sprekelsen et al. and Vilaseca et al. In one of them, they found that 94% of 401 consecutive disease-free patients treated with CO_2_TOLMS (including 254 T1–T2 glottic carcinomas) had a good QoL 1 year after treatment. Moreover, they found that older patients have a better perception of their disease. Considering that RT and neck dissection factor were associated with lower QoL, relative voice impairment was detected, especially in locally advanced tumor, and T classification (local early versus local advanced) and age were independent adverse prognostic factors for QoL within the speech domain [71]. In an earlier publication, 46% of patients considered speech as a relevant issue in QoL, while only 31% scored under reference values. In this paper, the author theorizes that the high expectations of minimal invasiveness created by CO_2_TOLMS and the considerable percentage of patients that continue working without any official recognition of their disability may explain this discrepancy [65]. Another publication by Valls et al. demonstrates that scores related with voice and swallowing outcomes remain consistent over time and do not deteriorate [72].

## 9. Complications

Complications in CO_2_TOLMS can be classified as intraoperative and postoperative (immediate or delayed). Also, complications can be divided into minor and major. Minor complications are those that can resolve spontaneously or those that can be treated in the office under local anesthesia, without any major consequences for the patient. Major complications are those requiring intensive medical treatment and even revision surgery [73].

Among the different types of complications that can affect patients, post-surgical bleeding is the most feared and the rate of this complication can be related with the site of the tumor, being more common in supraglottic tumors. Vilaseca et al. [73] reported 8% of bleeding in a series of 275 patients, of which 6.9% correspond to supraglottic tumors and 2.9% to glottic tumors. Similarly, Steiner and Ambrosch [74] reported a bleeding rate of 7% in supraglottic tumors and 0% in glottic tumors; whereas authors like Peretti et al. [75], Remacle et al. [76], and Canis et al. [77] reported 4%, 4.4%, and 9% of episodes of post-surgical bleeding from the treatment of supraglottic tumors. Bleeding may occur at home, where the possibility of fatal outcomes becomes higher [78]. Therefore, this complication should always be considered. For this reason, some authors advocate for prophylactic electrocoagulation of blood vessels at the laryngeal pedicle [78].

When CO_2_TOLMS is associated with neck dissection, some authors suggest ligation of the laryngeal branches of the external carotid artery [78]. Moreover, Ellies and Steiner, in a study that included 1528 patients treated for CO_2_TOLMS, showed a 4.7% (72 patients) incidence of post-surgical bleeding. External carotid ligation was required in 7 of such cases [79].

Regarding cervical complications after CO_2_TOLMS, Chiesa-Estomba et al. reported infectious cervical complications in 2 (2%) patients—one had cervical abscess formation and another one was complicated by mediastinitis [80]. Peretti et al. describe two cases of persistent cervical fistula after performing temporary tracheotomy in their patients [75]. Also, laryngeal vestibule stenosis after extensive laser resection of supraglottic tumor was described [77,80].

Motta et al. reported subcutaneous emphysema secondary to penetration of the cricothyroid membrane in 5% of patients, with it being necessary to perform a tracheotomy in four of them. Also, four patients required tracheotomy from edema, bringing the total amount of tracheotomies to 1% [3]. In another study performed by Eckel including 285 patients, one patient developed a glottic stenosis [81]. In another study performed by Vilaseca et al., 0.72% of cases developed thyroid cartilage chondritis after CO_2_TOLMS [73]. No other complications were seen.

## 10. Limitations of CO_2_TOLMS

Despite high rates of success in terms of oncological and functional outcomes described in the treatment of larynx lesions, CO_2_TOLMS has some limitations. Highlighted by Peretti, these limitations can be considered as the involvement of the posterior paraglottic space, the cricoarytenoid joint, or infiltration of the laryngeal framework negatively influencing oncological and functional outcomes [44].

An adequate laryngeal exposure is an important consideration when performing CO_2_TOLMS. To accomplish this, the laryngoscopy blade should be introduced looking for the best exposure and sometimes the resection of the ventricular fold is necessary when the exposure of the anterior commissure or the exposure of the laryngeal ventricle is not possible [82]. 

Another special situation is pretreatment reduced vocal fold mobility. In these cases, it will be important to ascertain whether this is due to tumor bulk, muscle infiltration, or fixation of the cryco-aritenoid because of the different prognosis of these situations. When the tumor affects the anterior commissure extending to the supra or subglottis in the cranio-caudal direction, a higher rate of local failure needs to be considered [82]. 

Nowadays, and thanks to Hartl’s reports, a better understanding about the function of the cricoid cartilage in the patent of the airway is considered. In his work, he demonstrated the risk of an unstable airway structure after cricoid cartilage resection and the risk of laryngeal stenosis and permanent tracheotomy. The need for cricoid cartilage resection should be stated as a contra-indication for CO_2_TOLMS. Also, to preserve laryngeal sphincter function, at least one cricoarytenoid unit (cricoid, arytenoid, cricoarytenoid joint/ muscles, and associated recurrent nerve) must be spared [83]. Finally, those patients in whom the vocal muscle or the arytenoid are affected will have a higher risk of recurrence [82]. 

Other limitations described that it has short penetration in tissues (0.1–0.3 mm on average), which can reduce the effective sealing of vessels (it coagulates vessels of calibers smaller than 0.5 mm), increasing bleeding during surgery [84,85]. Regarding the neck, a prophylactic treatment of the radiologically classified N0 neck in early glottis tumors is not necessary and the rate of occult neck metastasis in early glottic cancer is less than 10% [83].

## 11. Perspectives

### 11.1. Narrow Band Imaging 

CO_2_TOLMS glottic resections are usually considered oncologically acceptable when margins of 1 to 2 mm of healthy tissue are achieved. Therefore, the aim of any oncologic surgical treatment should be a complete tumor resection with adequate margins. However, the impact of positive margins after CO_2_TOLMS for glottic cancer on local control is still a matter of debate due to the large number of positives or inadequate margins not always clinically reflected in a corresponding high number of recurrences [56].

The introduction of new endoscopic devices, as well as the development of the “biologic endoscopy” concept [81], provides enormous value in defining tumor superficial extension. Among these diagnostic tools, narrow band imaging (NBI—Olympus Medical System Corporation, Tokyo, Japan) represents the more promising one: it consists of the use of filtered wavelengths that enhance the microvascular abnormalities associated with the preneoplastic and neoplastic changes of the mucosal lining of the upper aerodigestive tract [86,87,88].

Recently, Garafolo et al. reported their experience, using NBI in combination with a high definition screen to improve the pre- and intra-operative evaluation of patients, demonstrating that this a useful diagnostic tool to optimize the evaluation of the neoplastic boundaries as well as to reduce the incidence of positive superficial margins after CO_2_TOLMS [89].

### 11.2. New Laser Devices 

Due to the success of CO_2_ laser, new surgical tools based on other laser types are being developed. Some of these are KTP (potassium titanyl phosphate), Diode laser, PDL (pulsed dye laser), TH (Thullium), and Nd:YAG laser (Neodymium Yttrium Aluminum Garnet). The different laser devices use different wavelengths, affecting different chromophores of the tissue than the standard CO_2_ laser. The main advantage of this is increased preservation of the vocal fold soft tissue, including mucosal superior lamina propria (SLP), especially in superficial tumors leading to superior functional voice outcomes and a shorter wound healing period. 

In 2014, Zeitels published his results about the treatment of early glottic cancer (T1–T2) with the KTP laser. In this study, 117 patients were included and local control for T1 and T2 lesions was 96% and 80%, respectively. Further, 50% of recurrences were controlled with RT alone, while the other 50% failed RT and died of their disease. The author also reported a disease-specific survival of 99% for T1 and 89% for T2 [90]. This shows that at least in T1 carcinoma, KTP may be a valid alternative to traditional CO_2_ laser. However, the disease-specific survival in T2 was lower than that expected with CO_2_ laser. In 2014, Cömert showed that diode laser was effective compared with RT in patients with T1–T2 glottic carcinoma, reporting a local control with diode laser of 93% versus 90% for the group of patients treated by RT [91].

In 2007, Koufman et al. [92] reported their experience with multiple laser devices in office-based procedures, including 27 patients treated with Thulium laser. In 2017, Koss et al. published their results in the treatment of vocal fold leukoplakia through an office-based approach comparing the use of KTP versus PDL laser. Their results demonstrate the effectiveness of disease control with minimal morbidity and preservation of voice quality when using these two types of laser [93]. 

### 11.3. The Imaging as a Way to Improve Our Results

Ruytenberg et al. demonstrated the impact of Magnetic Resonance Imaging in the study of larynx cancer. This technique has gradually emerged as the preferred method of investigation due to its superior capability of space and tissue density resolution. New protocols of laryngeal radiofrequency coils and the application of respiratory-triggered acquisition techniques that minimize breathing and swallowing related artifacts and high-quality images (with an isotropic resolution of up to 1 mm) acquired in a few minutes will allow surgeons to obtain a set of nearly histopathologic macro-sections of the entire larynx, with a marvelous understanding of the 3D extent of the lesion to be treated [94]. 

### 11.4. A New Concept “Three-Dimensional (3D) Map of Isoprognostic Zones” 

A comprehensive approach to the categorization of different subgroups of glottic lesions to be treated by CO_2_TOLMS was described by Piazza et al. This classification system tries to overcome the intrinsic limitations of the contemporary edition of the TNM staging system in giving an affordable prognostication. The resulting 3D map of isoprognostic zones can effectively guide multidisciplinary teams in the management of a glottic tumor when a CO_2_TOLMS will eventually be performed, balancing alternative therapeutic options in the search for the best compromise between oncologic cure and functional results [95].

### 11.5. The Laser and the Robot 

Recently, it has been shown that combining Transoral Robotic Surgery (TORS) with a CO_2_ laser fiber, a precise cutting tool with a flexible tip, which is also an effective hemostasis of vessels 0.5 mm or less, is feasible [96]. Regarding the feasibility of TORS in T1–T2 carcinoma of the glottic and supraglottic larynx, it has been published preliminarily and has also been published recently [97]. However, the expectation is that in the future, TORS will be adapted for endolaryngeal surgery with better exposure and finer instruments. Recently, Chauhan et al. presented robotic microsurgical forceps for CO_2_TOLMS called the RMF-2F. This device offers a motorized tool-tip with forceps that open/close and rotate, which are able to improve precise motion, stabilize positioning, avoid hand tremors, reduce wrist excursions, and improve gesture scaling through teleoperation and improve safety through a tissue gripping haptic feedback [98].

### 11.6. Computer Assisted Laser Surgery of the Larynx 

The computer-assisted laser microsurgery (CALM) concept was recently presented by Deshpande et al. as a system to improve usability, efficiency, and controllability of the laser during surgery. This evolution of micromanipulators or hi-scan systems allows the surgeon to perform a surgery with improvements to ergonomics and accuracy, potentially improving surgical safety and quality. The system offers better use of the surgeon’s manual dexterity and intraoperative automated scanning of customized long incisions, achieving uniform resections at the surgical site. Moreover, the system offers automatic execution of intraoperative programmable scans combined with the high-speed scanning feature. Beyond this, it can potentially greatly reduce the amount of training required to achieve proficiency in CO_2_TOLMS [99].

### 11.7. The Laryngoscore 

In order to improve the preoperative assessment of patients before CO_2_TOLMS, in 2014, Piazza et al. presented an instrument called "The Laryngoscore", including 11 parameters: interincisors gap, thyromental distance, upper jaw dental status, trismus, mandibular prognathism, macroglossia, micrognathia, degree of neck flexion-extension, history of previous open-neck and/or radiotherapy of the head and neck, Mallampati’s modified score, and body mass index, demonstrating its reliability and reproducibility, allowing surgeons to identify good laryngeal exposure with high confidence, while also identifying possible difficult laryngeal exposure [100].

## 12. Conclusions

CO_2_TOLMS is a safe and effective option as an organ preservation strategy in the treatment of early glottic carcinoma (Tis–T2), as it is associated with less morbidity and a high percentage of local control, both overall and specific to survival. Oncological outcomes can be influenced by some situations like anterior commissure or arytenoid involvement. However, relevant new perspectives suggest an exciting future for this technique in the upcoming years. 

## Figures and Tables

**Table 1:** Endoscopic cordectomy classification of the European laryngological society.

Type of Cordectomy	Type of Lesion	Target (Diagnostic or Therapeutic).
**I**	Limited to the epithelium: benign lesions or a Ca. In Situ without signs of microinvasion	Can be Diagnostic or Therapeutic
**II**	Microinvasive carcinoma	Therapeutic
**III**	Small superficial cancer of the mobile vocal fold reaching the vocal muscle without deep infiltration	Therapeutic
**IV**	Almost a T1a cancer with Deep infiltration of the vocal muscle.	Therapeutic
**Va**	Malignant lesion of the vocal fold superficially reaching the anterior commissure without infiltrating it	Therapeutic
**Vb**	Malignant lesion invading the arytenoid without fixation of the crycoaritenoid articulation	Therapeutic
**Vc**	Ventricular or transglottic malignant lesion spreading from the vocal fold to the ventricle	Therapeutic
**Vd**	Glottic malignancy with limited spread from the vocal cord to the subglottis	Therapeutic
**VI**	A lesion from the anterior commissure without infiltration of the thyroid cartilage	Therapeutic

**Table 2:** Benign, acquired, and premalignant lesions of the larynx able to be treated with CO_2_TOLMS.

Benign, Acquired, and Premalignant Lesions of the Larynx	
**Congenital Lesions**	- Laryngomalacia - Laryngeal cysts - Subglottic hemangioma - Vocal fold paralysis - Congenital subglottic stenosis
**Acquired Lesions**	- Nodules - Polyps - Cysts - Reinke Edema - Post-intubation granuloma - Post-radiotherapy arytenoid edema - Laryngeal papillomatosis - Amyloidosis - Bilateral vocal fold paralysis - Posterior cordectomy - Laryngeal stenosis
**Premalignant Lesions**	- Leukoplakia - Keratosis - Hyperplasia
